# Crystal growth kinetics as an architectural constraint on the evolution of molluscan shells

**DOI:** 10.1073/pnas.1907229116

**Published:** 2019-09-24

**Authors:** Vanessa Schoeppler, Robert Lemanis, Elke Reich, Tamás Pusztai, László Gránásy, Igor Zlotnikov

**Affiliations:** ^a^B CUBE–Center for Molecular Bioengineering, Technische Universität Dresden, 01307 Dresden, Germany;; ^b^Institute for Solid State Physics and Optics, Wigner Research Centre for Physics, 1525 Budapest, Hungary;; ^c^Brunel Centre of Advanced Solidification Technology, Brunel University, UB8 3PH Uxbridge, Middlesex, United Kingdom

**Keywords:** biomineralization, molluscs, solidification, crystal growth, morphogenesis

## Abstract

Molluscan shells are a classic model system to study formation–structure–function relationships in biological materials and the process of biomineralized tissue morphogenesis. Typically, each shell consists of a number of highly mineralized ultrastructures, each characterized by a specific 3D mineral–organic architecture. Surprisingly, in some cases, despite the lack of a mutual biochemical toolkit for biomineralization or evidence of homology, shells from different independently evolved species contain similar ultrastructural motifs. In the present study, using a recently developed physical framework, which is based on an analogy to the process of directional solidification and simulated by phase-field modeling, we compare the process of ultrastructural morphogenesis of shells from 3 major molluscan classes: A bivalve *Unio pictorum*, a cephalopod *Nautilus pompilius*, and a gastropod *Haliotis asinina*. We demonstrate that the fabrication of these tissues is guided by the organisms by regulating the chemical and physical boundary conditions that control the growth kinetics of the mineral phase. This biomineralization concept is postulated to act as an architectural constraint on the evolution of molluscan shells by defining a morphospace of possible shell ultrastructures that is bounded by the thermodynamics and kinetics of crystal growth.

## Significance

Using notions from classic materials science, we expand our understanding of the macroscopic morphospace of possible molluscan shell shapes to the level of possible ultrastructures that comprise them. This provides us with a unique opportunity to explore this morphospace using well-developed analytical, theoretical, and numerical tools and to test the effects of a discrete number of parameters on shell biomineralization. The physical model presented here sheds a new light on the evolutionary aspect of molluscan shell ultrastructural fabrication and suggests that the repeated “discovery” of some mineral morphologies partially reflects a series of architectural constraints provided by biomineral growth kinetics.

Molluscan shells exhibit a diverse range of mineral–organic composite ultrastructures, many of which originated during the early Paleozoic, starting with the first mineralized shell appearing during the Cambrian (1). These shells perform several functions, which include encapsulating the body, separating the inner soft tissue from the environment, and providing mechanical protection from predators (2–4). The mechanical performance of these shells is strongly dependent on the 3D organization of these biocomposites, which in many cases provide enhanced strength and toughness compared to the pure mineral phase and superiority compared to modern man-made composites (5, 6). As a result, molluscan shells are a classic model system to study formation–structure–function relationships in biological materials and the process of biologically controlled mineral formation.

Typically, molluscan shells consist of a number of layers that lie parallel to the outer shell surface. Each layer is characterized by a specific shell ultrastructure (e.g., prismatic, lamellar, spherulitic, and nacreous) and a specific calcium carbonate polymorph, aragonite or calcite (7–9). Surprisingly, in many cases, shells from different independently evolved species and even different classes (i.e., gastropods, cephalopods, bivalves, and monoplacophorans) contain similar shell ultrastructures (10). Moreover, transcriptomic and proteomic analyses of shell-depositing tissues of various species comprised of similar shell ultrastructures revealed some basic molecular functions, such as protease inhibition or melanin formation, that seem to be recurrently present in the different models (11). However, so far, hardly any similarities were found in molecular repertoires that are responsible for biomineral fabrication (10, 12–15). Yet, the physiological principle of shell biomineralization is highly conserved among molluscs. It is postulated that shell formation is an extracellular process where a layer of specialized cells in the mantle epithelium secrets a complex mixture of organic and mineral precursors into a confined space (i.e., the extrapallial space) that is located between the outer organic layer (i.e., the periostracum) and internal mantle tissue (16). Whereas the existence of this space and its size are still under debate, the majority of experimental evidence points toward its crucial role in shell biomineralization (17). Here, the different ultrastructures were hypothesized to form via self-assembly and grow in thickness from the periostracum toward the mantle cells, which guide their morphogenesis by changing the physical boundary conditions (e.g., saturation level, pH, and viscosity) and the chemistry of the solidifying medium by using a repertoire of organic and inorganic precursors (8, 18–20). For example, specific interactions of biomolecules with mineral precursors were shown to affect nucleation, polymorph selection, crystallization pathway, and the growth process of the mineral phase (21–25). However, no biochemical toolkit for the formation of a specific ultrastructure has been found so far (26). Although a few models that explain the generation of some morphologies, such as the nacreous (27, 28) and the prismatic layer (18) exist, the exact mechanisms by which the cells control the formation of the various ultrastructures and the transition from one biocomposite architecture to another remain unclear.

Recently, a physical model with the capacity to describe the formation of the entire aragonitic shell of the bivalve *Unio pictorum*, which consists of 3 different ultrastructures, was introduced (20). By drawing an analogy to the concept of directional solidification, well known in the field of materials science (29, 30), the ability of the model to fully describe the morphogenesis of the entire shell construct on the ultrastructural and nanostructural levels was demonstrated. Structural development of the shell in thickness was shown to be the result of a transition from a fast to a slow directional solidification mode, accompanied by an increased morphological regularity. This process was hypothesized to be orchestrated by the cellular tissue, by reducing the concentration of the mineral precursor in the extrapallial space and, thus, reducing the driving force for solidification. In the present work, we studied the purely aragonitic shells of 2 species from 2 molluscan classes: the cephalopod *Nautilus pompilius* and the gastropod *Haliotis asinina*. Both shells exhibit a continuous gradual transition from a granular to a regular columnar to a highly ordered columnar nacreous ultrastructure. The presented structural analysis is fully consistent with the developed directional solidification model (20), which similarly to the bivalve *U. pictorum*, suggests that the morphogenesis of the different ultrastructural layers and the transition between them is a result of a progressively decelerating solidification process. Thus, we demonstrate that the introduced model is comprehensive and can describe the process of formation and independent evolution of a variety of ultrastructures in various molluscan classes despite the lack of a common biochemical toolkit for biomineral morphogenesis. Furthermore, we show that the fabrication of these biocomposites is controlled by the organisms by regulating the growth kinetics of the mineral phase, which is suggested to be key in determining the morphospace of possible shell ultrastructures and, therefore, acts as an architectural constraint on the evolution of molluscan shells.

## Results

### The Shell of *N. pompilius*.

**Fig. 1.**
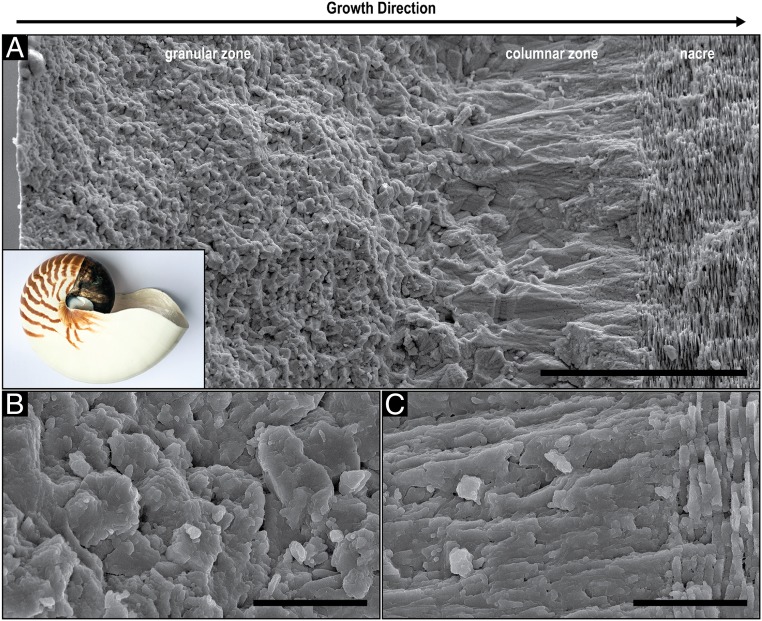
Structural analysis of the *N. pompilius* shell using electron microscopy. (*A*) SEM image of a fractured cross-section of the shell of *N. pompilius* prepared perpendicular to the shell’s outer surface exposing the granular, columnar and nacreous layers (*Inset* shows the entire shell). (Scale bar, 50 µm.) (*B*) Higher magnification of the central region of the granular layer. (Scale bar, 5 µm.) (*C*) Higher magnification of the columnar–nacre transition. (Scale bar, 5 µm.)

The shell wall of *N. pompilius* is commonly divided into 3 layers: The outer prismatic and nacre layers (Fig. 1*A*) and the inner prismatic layer. Scanning-electron microscopy (SEM) images of a fractured surface reveal that the outer prismatic layer exhibits 2 different morphologies. Initially, the shell is composed of micrometer-sized granules (granular zone), which gradually increase in size along the direction of growth before they transform into columnar units (columnar zone), several tens of microns long, which fan out toward the next layer, nacre. Higher magnifications of the granular layer and the columnar-to-nacre transition (Fig. 1 *B* and *C*, respectively) show the typical nanoparticle substructure (31). Fig. 1*C* depicts the gradual transition between the columnar ultrastructure and nacre, which consists of ∼300-nm-thick tablets.

**Fig. 2.**
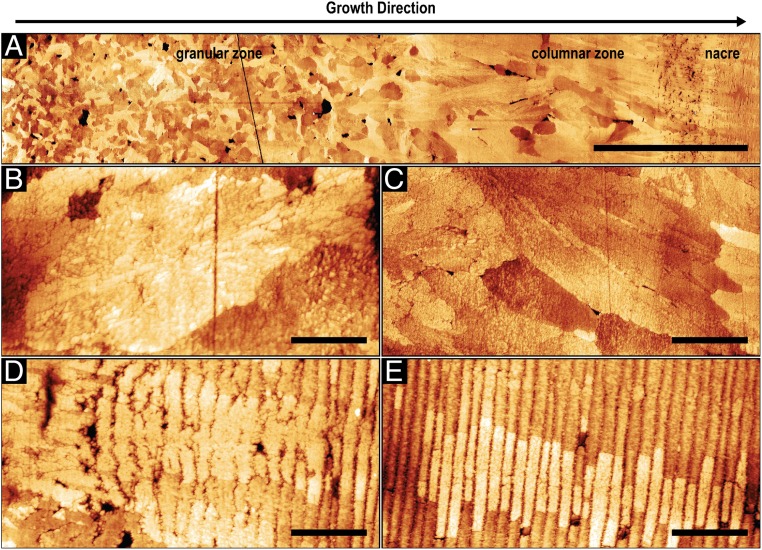
Structural analysis of the *N. pompilius* shell using AFM. (*A*) AFM map of a polished cross-section prepared perpendicular to the shell surface showing the transition from the granular to columnar to the nacreous layer. (Scale bar, 50 µm.) (*z*-range, 75 nm.) Higher magnification AFM maps of: (*B*) a single granule in the granular zone, (*C*) a prism in the columnar zone, (*D*) the columnar to nacre transition, and (*E*) well-developed nacre. (Scale bars: 2 µm, 4 µm, 2 µm, and 2 µm, respectively.) *z*-ranges are 40 nm, 39 nm, 42 nm, and 38 nm, respectively.

Atomic force microscopy (AFM) measurements also demonstrate the different morphologies of the shell and provide additional information on the substructure of the mineral units (Fig. 2*A*). Mechanical polishing led to minor height differences between the individual mineral blocks, probably due to their different crystallographic orientations and, thus, different abrasion efficiencies. In Fig. 2*B*, a representative mineral unit of the central region of the granular ultrastructure is shown demonstrating that it has a spherulitic nature. The granule is ∼10 µm long and 4 µm wide and a nanometer-sized substructure that radiates from its center is recognizable. However, besides slight differences in height, no clear boundary with adjacent mineral blocks that would indicate a presence of an organic envelope around the granule is visible. Similarly, the columnar units show no distinct organic interfaces (Fig. 2*C*). In contrast, clear boundaries between nacre tablets where the interlamellar organic sheets are located are resolved (vertical lines in Fig. 2 *D* and *E*). Comparing the structure of nacre tablets directly at the transition zone (Fig. 2*D*) with nacre tablets located ∼30 µm from the transition (Fig. 2*E*), we find that differences in regularity, the shape, and the spacing between the tablets are distinct. Directly after the transition the thicknesses of the lamella vary between 100 nm and 500 nm and the interlamellar boundaries are corrugated and partially diffuse, leading to occasional intergrowth of superimposed layers. In contrast, the thickness of the tablets in the main body of nacre is regular. Here, the interlamellar membranes form straight lines and clearly separate the individual layers. In both cases, no horizontal boundaries separating the different tablets in a single layer are visible, indicating a much lower amount of organic material between the nacre tablets compared to the interlamellar membranes. In addition, the crystallographic orientation of the tablets seems to be inherited from the underlying tablets (32), as it is evident from similar height profiles of tablets stacked parallel to the direction of growth, from left to right in Fig. 2*E*.

**Fig. 3.**
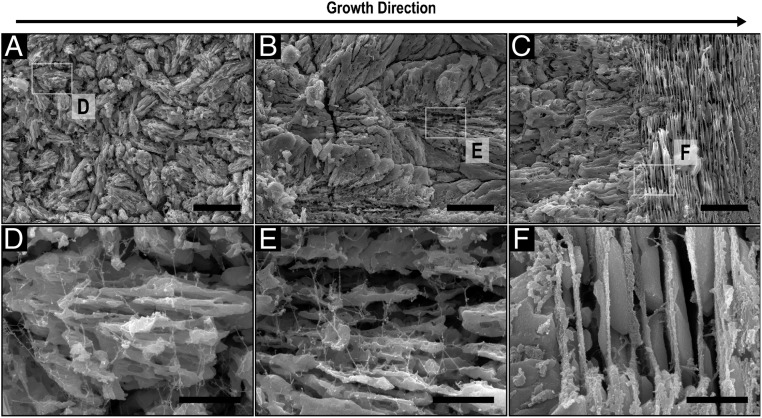
Organic components in the shell of *N. pompilius*. SEM images of a cross-section that was cut perpendicular to the shell’s surface and etched for 2 min with 2% EDTA showing: (*A*) the granular zone, (*B*) the columnar zone, and (*C*) the columnar to nacre transition. (Scale bars, 5 µm.) (*D*–*F*) Higher magnifications of *A*–*C*, respectively. (Scale bars, 1 µm.)

To investigate the distribution of organic components and to better differentiate the individual mineral units, a cross-section of the shell was slightly etched with EDTA (Fig. 3). After etching, the boundaries appear more distinct and a branched dendritic-like substructure of the mineral blocks in the granular and the columnar zones is revealed (Fig. 3 *A* and *B*, respectively). In agreement with the previous data, no organic envelopes surrounding the different units are visible. Approximately 10-nm-thick fibers are evident in both layers. They seem to be randomly distributed and interconnected within and between the mineral phase (Fig. 3 *D* and *E*). In addition, globular entities with a diameter of ∼35 nm are seen to be attached to these filaments. Similar structures were found in the prismatic layer of the bivalve *Atrina rigida* and were identified as disordered chitin fibers with attached proteins (33). In the columnar-to-nacre transition zone (Fig. 3 *C* and *F*), the organic matter appears to be agglomerated and only few isolated fibers are visible. In nacre, the interlamellar matrix, previously reported to contain crystalline chitin (27, 34), is visible as rough sheets ∼40 nm in thickness with a few isolated fibers connecting the adjacent membranes (Fig. 3*F*). The mineral tablets appear to be dense and structurally homogeneous compared to the units in the other 2 ultrastructures and no distinct organic membranes segmenting the tablets in the same nacre layer are visible.

**Fig. 4.**
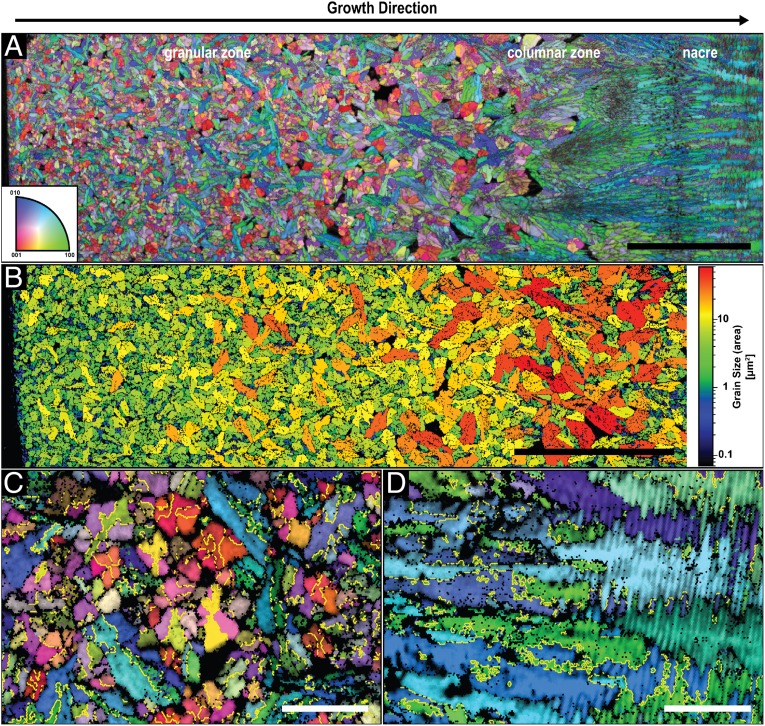
Crystallographic analysis of the shell of *N. pompilius*. (*A*) EBSD map of a polished cross-section prepared perpendicular to the outer surface of the shell. The corresponding color-coded inverse pole figure of aragonite, with the reference direction normal to the image plane, is depicted in the *Inset*. (Scale bar, 50 µm.) (*B*) Mineral units size map of the granular zone of the map in *A*. Single granules were identified using a tolerance angle of 5° while taking the twin boundaries into consideration. (Scale bar, 50 µm.) (*C* and *D*) Higher-resolution EBSD map of the granular zone and the columnar–nacre transition, respectively, with the same color coding as in *A*. The yellow lines indicate the typical {110} aragonite twin boundaries, which were calculated by identifying misorientation angles between the {110} planes of 64° ± 5°. (Scale bars, 5 µm.)

Electron backscatter diffraction (EBSD) measurements were performed to analyze the crystallographic characteristics of the studied shell ultrastructures. An EBSD map of the granular and the columnar layer and the transition to nacre is presented in Fig. 4*A*. EBSD confirms that the entire shell is aragonitic (9). In the granular zone, every mineral unit exhibits a single-crystal–like nature. Whereas initially, the small granules show no preferred orientation, a gradually increasing size (Fig. 4*B*) and coalignment of the *c*-axis of aragonite with the direction of growth (Fig. 4*A*) are observed until the columnar zone, where high level of texture is obtained. Here, the *c*-axis of aragonite in all of the mineral units is parallel to the direction of growth. The nacre tablets inherit their crystallographic orientation from the underlying columnar assembly and continue to grow maintaining the preferred orientation (Fig. 4*A*).

In Fig. 4*C*, typical aragonite twinning on {110} planes and a misorientation angle of ∼64° is marked by yellow lines showing that the majority of the granules are twinned crystals. Interestingly, granules with their *c*-axis of aragonite being almost perpendicular to the image plane (red colors) demonstrate the classic cyclical twinning of aragonite (35–37). They exhibit 6 crystallographic domains fanning out from the center of the crystal in which pairs of domains with a similar orientation are located opposite to each other. The granules with the *c*-axis of aragonite parallel to the growth direction (blue and green colors) are elongated in shape and the twin boundaries follow their long axes. At the transition to nacre (Fig. 4*D*), the columnar units gradually transition into the nacreous layer while maintaining the crystallographic orientations of the mineral and keeping its twinned characteristics.

### The Shell of *H. asinina*.

**Fig. 5.**
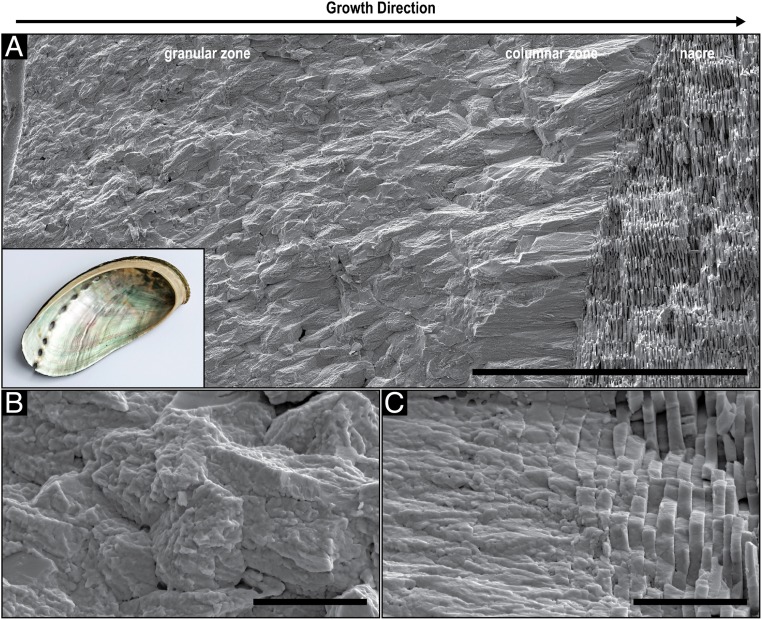
Structural analysis of the *H. asinina* shell. (*A*) SEM image of a fractured cross-section of the shell of *H. asinina* prepared perpendicular to its outer surface showing the granular, columnar and nacreous layers (*Inset* shows the entire shell with the nacreous layer exposed). (Scale bar, 100 µm.) (*B*) Higher magnification of the initial granular layer. (Scale bar, 5 µm.) (*C*) Higher magnification of the columnar–nacre transition. (Scale bars, 5 µm.)

Fig. 5*A* shows an SEM image of a fractured shell of *H. asinina*, demonstrating its 3 ultrastructural motifs. Similar to *N. pompilius*, the morphology of the mineral phase gradually changes from a granular zone to a columnar zone to a nacreous ultrastructure along the direction of growth, and the mineral building blocks exhibit a nanoparticle substructure (Fig. 5 *B* and *C*). Nevertheless, the mineral units in the granular zone and the thickness of the tablets in nacre are larger than in *N. pompilius*.

**Fig. 6.**
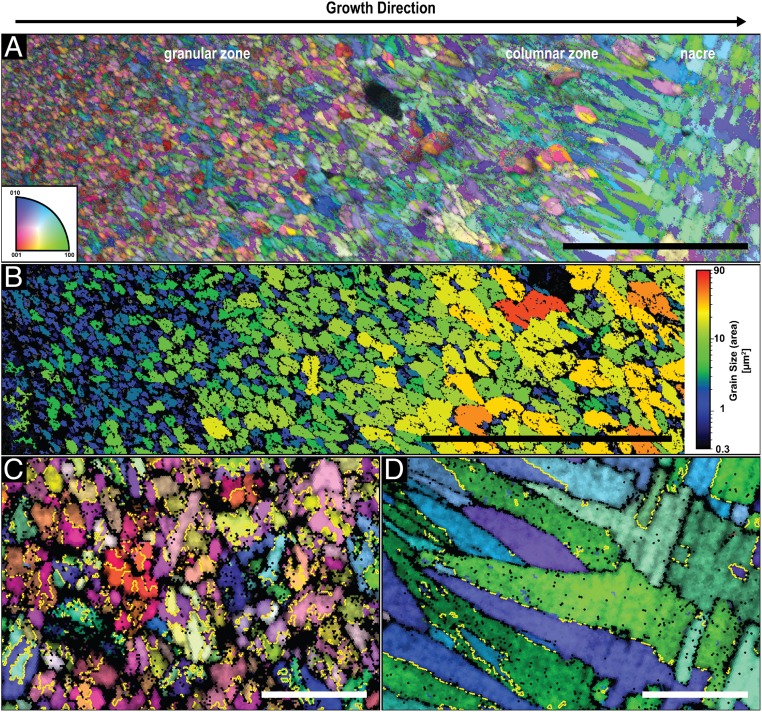
Crystallographic analysis of the shell of *H. asinina*. (*A*) EBSD map of a polished cross-section prepared perpendicular to the shell’s surface. The corresponding color-coded inverse pole figure of aragonite, with the reference direction normal to the image plane, is depicted in the *Inset*. (Scale bar, 50 µm.) (*B*) Mineral units size map of the granular zone of the map in *A*. Single granules were identified using a tolerance angle of 5° while taking the twin boundaries into consideration. (Scale bar, 50 µm.) (*C* and *D*) Higher-resolution EBSD map of the granular zone and the columnar–nacre transition, respectively, with the same color-coding as in *A*. The yellow lines indicate the typical {110} aragonite twin boundaries, which were calculated by identifying misorientation angles between the {110} planes of 64° ± 5°. (Scale bars, 5 µm.)

EBSD analysis of a polished *H. asinina* shell, displayed in Fig. 6*A*, confirms that this shell is also exclusively aragonitic (38). In addition, similar to *N. pompilius*, the individual mineral units are twinned single crystals (Fig. 6 *C* and *D*) that increase in size with the direction of growth (Fig. 6*B*). In the granular zone, the typical cyclical twinning is visible in spherulites having their {001} planes of aragonite parallel to the image plane (red colors in Fig. 6*C*). Whereas, initially, most of the granules are randomly oriented, a gradual preferred orientation is developed and the *c*-axis of aragonite slowly coaligns with the direction of growth (Fig. 6*A*). In the columnar zone, the mineral units almost exclusively have the *c*-axis of aragonite oriented parallel to the growth direction and along the long axis of the columns (Fig. 6*A*). The transition to nacre is also gradual and the crystallographic properties of the tablets are inherited form the columnar ultrastructure (Fig. 6*D*).

## Discussion

### Thermodynamic and Kinetic Aspects.

Detailed analysis of the shells of *N. pompilius* and *H. asinina* reveals high structural and crystallographic similarities between the cephalopod and the gastropod shells. In both species, the investigated mineralized layers are solely aragonitic and are currently considered as 2 individual layers that where classically described as a prismatic and a nacreous layer (39, 40). However, by the end of the 19th century, researchers observed radiating crystalline structures in the first mineralized layer of *N. pompilius* (41) and a prismatic sublayer close to the beginning of nacre (42). Mutvei (43) described it as a spherulitic-prismatic layer. In this work, we confirm this observation in *N. pompilius* (Fig. 1*A*) and in *H. asinina* (Fig. 5*A*). Traditionally, the spherulitic-prismatic structure in *N. pompilius* was considered as a single layer. This interpretation is consistent with the presented results since the granular ultrastructure transitions gradually into the prismatic ultrastructure in both species. Moreover, the transition from the prismatic into the nacreous ultrastructure is gradual as well (Figs. 1*C* and 5*C*). This indicates that the different ultrastructures in these shells, including the nacre, are not truly individual, divisible layers, but rather one continuous construct formed following a common growth mechanism.

Recently, it was demonstrated that the ultra- and the nano-structural evolution of the bivalve shell of *U. pictorum* can be described by the process of directional solidification (20), an extensively studied concept from material science that is used to elucidate how materials solidify along thermal and concentration gradients (30). Specifically, the morphogenesis of the entire aragonitic shell construct exhibiting a gradual transition from a dendritic, to a prismatic, and ultimately to a nacreous ultrastructure was explained from the view point of crystal growth thermodynamics and kinetics. In general, pattern formation during directional solidification is guided by capillarity and diffusion processes, which are steered by the thermodynamic driving force for solidification (e.g., temperature and/or supersaturation levels) (30, 44). In the case of *U. pictorum*, the hypothesized driving force for shell morphogenesis is the level of mineral precursor concentration in the solidifying medium, which was evident in an increasing degree of structural order. This indicates a transition from a fast to a slow growth mode, which was suggested to be induced by a decreasing mineral precursor concentration in the extrapallial space. Similar to *U. pictorum*, the shells of *N. pompilius* and *H. asinina* are deposited continuously in a directional manner and show an increasing degree of order that is apparent on the ultrastructural level. Here, the granular morphology demonstrates a gradual increase in size (Figs. 4*B* and 6*B*) until the granules morph into the columns of the prismatic zone and, finally, into the highly ordered nacreous structure (Figs. 1*A* and 5*A*). Moreover, this process is followed by a gradual crystallographic texturing (Figs. 4*A* and 6*A*).

Biologically controlled biomineralization is a form of heterogeneous nucleation where new crystal formation is induced by cells on a surface or in solution on impurities or particles, such as biomolecules (45–48). During directional solidification, the addition of impurities/particles (nucleation centers) to a solidifying liquid in combination with relatively high solidification driving forces can cause equiaxed growth, which is defined by the formation of new randomly oriented globular or dendritic grains ahead of the growth front (49–51). The size and shape of the grains is directly correlated with the amount of nucleation centers (52) and the solidification velocity (53). Higher concentrations of nucleation centers and growth front velocities lead to higher numbers of smaller grains, whereas at lower concentrations and lower velocities the grains increase in size until they transition into columns (52, 53). Here, the direction of grain elongation is controlled by the applied gradient of the driving force (54). Occurrences that strongly affect the final morphology of a solidifying material are segregation events, which are also caused by additives in the solidifying fluid. Segregation leads to local chemical inhomogeneities that can manifest as inclusions between the dendrite arms, and between more complex structures, such as columnar and banded morphologies (55). Segregation-induced structuring depends mainly on the solidification velocity and the size of the additives (56): At slow velocities, they are more likely to be repelled and at faster velocities, the smaller additives are more likely to be entrapped in the structure.

The ultrastructures observed in the *N. pompilius* and the *H. asinina* shells show strong analogies to morphologies that occur during directional solidification processes, and the described structural transformations indicate a decreasing solidification velocity during shell formation. The appearance of individual, randomly oriented granules in the first shell layer (Figs. 4*A* and 6*A*) indicates a heterogeneous nucleation mechanism that can potentially be induced by any kind of a biomolecular complex secreted by the mantle cells. The granules grow as single crystals in a branched manner typical for the equiaxed solidification mechanism (Figs. 2*B* and 4*C*), where they increase in size until they impinge on each other and block further growth (57, 58). During further shell growth, the nucleation rate and growth kinetics change, which is reflected in the increasing granule sizes and gradual emergence of texture (Figs. 4 and 6). In molluscs, this can be accomplished by a decrease in mineral precursor and nucleation center concentrations. It is important to note that another factor that can influence the driving force for solidification is components in the extrapallial fluid that bind or chelate calcium ions. Many molluscan shell proteins exhibit calcium binding domains (11, 59) and are suggested to affect the pathway of CaCO_3_ crystallization (25). Moreover, other ingredients in the extrapallial fluid, such as chelating amino acids or carbohydrates, bind calcium (60) and, thus, can affect crystallization rates. However, once the driving force for solidification is low enough, sustained grain growth becomes predominant and new nucleation events become less likely and eventually, cease.

The {001}-planes of aragonite have the lowest attachment energy and, hence, an aragonite crystal is expected to grow the fastest along its crystallographic *c*-axis (61). Indeed, this differential growth is observed in the granular zone of *N. pompilius* (Fig. 4) and *H. asinina* (Fig. 6). In a slower growth rate regime where growth front nucleation has stopped, the faster growing faces are sustained due to competition along the growth direction and, similar to what happens during classic directional solidification, columns are formed (54). In both species, this process is followed by an increase in crystallographic texture (Figs. 4 and 6). However, it is important to note that other parameters can also induce growth anisotropy. As previously stated, common factors that affect morphogenesis during crystal growth include temperature, supersaturation, additives, and viscosity of the solidifying medium (62–66). Specifically, biogenic crystal growth kinetics can be influenced by biomolecules, such as proteins, by binding to specific crystal planes and thus, enhancing or inhibiting their growth. In molluscan shells, different binding motives, such as acidic proteins, have been identified to influence final crystal shapes during shell development (67–69). Thus, we cannot rule out that a complex combination of various factors, as for example, a decrease in mineral concentration together with the addition of biomolecules and a general decrease in viscosity, affect growth anisotropy and thereby texture.

The distribution of the organic components in shell layers is also consistent with a reduced growth rate hypothesis. The organic phase, which is mainly visible as chitin with attached proteins (Fig. 3 *D* and *E*) (20, 33, 70), is initially distributed within and between mineral units without forming distinct structures. As mentioned above, during solidification at very fast growth velocities, solute trapping is most likely leading to equal distribution of the organic components within and between the minerals. Closer to nacre, considerably less of the organic matter is entrapped and is instead located between the mineral blocks in a more consolidated form (Fig. 3*F*), which indicates a reduced solidification rate and a possibly decreased mineral to organic ratio. Similar to *U. pictorum* (20), the gradual transition into nacre indicates that the solidification front has reached a steady-state regime and its velocity is low enough to allow chitin crystallization (34) ahead of the mineralizing front. Finally, nacre formation is enabled in a way that is consistent with classical models for the solidification of banded microstructures (71, 72) and current models for nacre morphogenesis (27).

An additional indication of a very fast growth mode during the formation of initial shell layers in *N. pompilius* and *H. asinina* is the existence of twins (Figs. 4 *C* and *D* and 6 *C* and *D*). Twinning commonly occurs during crystallization and is frequently observed in solidification processes (73, 74). There are several conditions that can cause twinning, some of which can happen after initial crystallization, such as gliding twinning or transformation twinning (75). Twins observed in mollusc shells are growth twins, which develop during the nucleation stage and are typically induced by very high levels of supersaturation and, hence, high crystallization velocities, and by organic impurities, such as proteins (76, 77). In *N. pompilius* and *H. asinina*, cyclic {110} twinning that typically occurs in biogenic (78) and abiotic (79) aragonite is observed in all randomly oriented mineral units (Figs. 4*C* and 6*C*), again confirming the hypothesis of self-assembly. Furthermore, twinning contributes to a faster shell formation since it is more effective than nontwinned growth forms in terms of space filling (80).

### Phase-Field Modeling.

To illustrate that directional solidification represents a reasonable concept for the formation of the 2 studied shells, we performed phase-field simulations of their ultrastructural morphogenesis using an orientation field-based model (81). Here, we adopted a regular solution thermodynamics that realizes a hypothetical quasi-binary eutectic system. This approach allows simultaneous precipitation of 2 solid phases with different compositions: A mineral-rich phase and another that is rich in organic matter, whereas the extrapallial fluid was assumed to be a solution of both. It is important to note that only qualitative phase-field simulations are possible at this stage as quantitative simulations would require a considerably more detailed knowledge of the system, including the thermodynamic properties of the essential constituents, the diffusion coefficients of the relevant species, and the free energies for the solid–liquid and solid–solid interfaces. However, our minimal model appears to be a useful working approximation.

In compliance with the hypothesis that the ultrastructural evolution in *N. pompilius* and *H. asinina* shells can be accomplished by a gradual decrease in the concentration of the mineral precursors (driving force for solidification) and nucleation centers (heterogeneous nucleation mechanism), the following assumptions were made. 1) The equiaxed granules form via heterogeneous nucleation on randomly oriented particles (nucleation centers), whose density decreases exponentially with the distance from the inner surface of the periostracum. These particles were represented by single pixel domains bound by surfaces of the same wetting properties as the bulk periostracum. The wetting properties were set by a boundary condition described in previous works, “Model A” in refs. 82 to 84. 2) The thickness of the extrapallial space was kept constant. This assumption was ensured by a moving boundary condition at the outer surface of the mantle, which was moved synchronously with the growing solid phases. Finally, it was assumed that 3) at this surface the concentration of the mineral phase decreased exponentially with time.

**Fig. 7.**
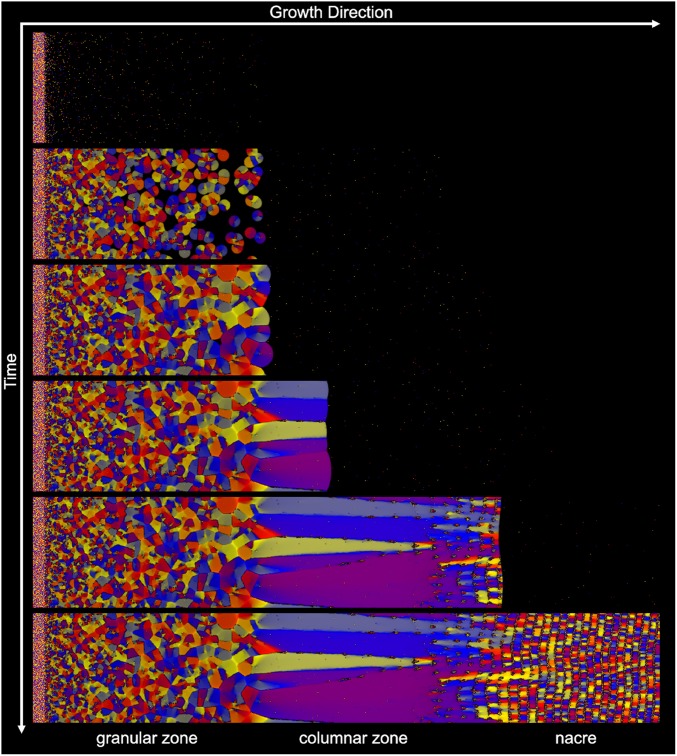
Formation of a shell as predicted by the orientation field based phase-field simulation. Time increases from *Top* to *Bottom* showing increasingly mature growth stages of the shell. The periostracum is the thin disordered layer on the left. Solidification starts with heterogeneous nucleation on small nucleation centers, whose density decreases from left to right exponentially. At the same time, due to the decreasing level of mineral supersaturation nucleation is ceased entirely and elongated columnar crystals are formed. This is followed by the formation of a banded structure via a previously described mechanism (85).

The evolution of the microstructure predicted under these conditions is shown by a sequence of snapshots taken during the simulation (Fig. 7) and by animations presented in *SI Appendix* (Movies S1–S3). The results appear to be in a remarkable qualitative agreement with the EBSD maps collected from both species (Figs. 4*A* and 6*A*). First, a rapid formation of an equiaxed structure made of small randomly oriented grains is observed. The decreasing density of nucleation centers results in a decreasing nucleation rate and a grain size that increases toward the mantle and, eventually, leads to the formation of a columnar microstructure. In parallel, the growth rate decreases with the decreasing driving force and a 2-phase solidification becomes more pronounced. This leads to the formation of alternating mineral-rich and organic phase-rich layers [analogously to the behavior predicted by phase field simulation for eutectic solidification at high undercooling (85)], and gives rise to a morphology closely resembling that of the nacre. In fact, even the mineral bridges that traverse the organic layers and are suggested to transmit the crystallographic orientation between successive mineral layers in nacre (86) are reproduced by the model (Movie S2).

### Biochemical and Genetic Aspects.

It is well recorded that granular spherulitic structures solidify quickly and that they are found in many different tissues that are known for rapid mineralization (21). Spherulitic structures frequently appear in the early stages of shell development of bivalve, gastropod, and cephalopod larvae (87–90) and during shell repair (91, 92). Newly formed granular aragonitic structures, as it was shown for paralarvae and a repaired shell of *Nautilus macromphalus* (89, 91), often transition into a prismatic ultrastructure and later into nacre, and thus, resemble the regular structure of the shell. However, in some cases, they strongly differ from it. For example, the adult shell of *Pinctada margaritifera* (bivalve) exhibits separated layers of calcitic prisms and aragonitic nacre but the initial larvae shell is spherulitic–prismatic and exclusively aragonitic (87). The occurrence of the spherulitic–prismatic ultrastructure in different species and classes when a rapid shell growth is presumably necessary; that is, the formation of initial protection during embryonic development, indicates a functional constraint on the expedient growth of a calcium carbonate shell.

Interestingly, during shell repair, the same cells that so far have been producing one specific ultrastructure (e.g., nacre) are able to switch to another faster growth form, which very often is the spherulitic–prismatic ultrastructure (91, 92). Based on gradual transitions between the different morphologies—and in accordance to the directional solidification model—Fleury et al. (92) suggested that this does not require major changes in the molecular composition of the solidifying medium but a rather minor adjustment in the proportions between the existing components. However, little is known about the organic composition and its effect on molluscan shell ultrastructural morphogenesis and, therefore, the study of the biochemical composition of the shells (e.g., matrix proteins) in a variety of organisms is a major interest in the field (11, 93). Different protein compositions were found in the various shell ultrastructures in *Pinctada* species (94), which are composed of 2 distinct layers: Calcitic prisms and aragonitic nacre. In contrast, the proteins found in all layers of *U. pictorum* and *H. asinina*, both showing a gradual transition between their exclusively aragonitic ultrastructures, are highly similar (39, 95). In addition, elemental analysis revealed no significant differences in magnesium, strontium, sodium, and sulfur content in the different ultrastructures of *H. asinina* (38). In fact, in congeneric species (11, 39, 94), as well as in species from different classes, homologous proteins related to the biomineralization process are extremely rare regardless of the similarity in the ultrastructural assembly they comprise (96). One of the best examples in this context is nacre, which evolved independently in bivalves, gastropods, cephalopods, and monoplacophorans between the Middle Cambrian and Lower Ordovician (97). This explains the high number of heterologous proteins found in molluscan shells and possibly indicates that the emergence of a specific ultrastructure is not guided by a specific molecular machinery. Why then do these mineralizing systems show the repeated “discovery” of similar biocomposite ultrastructures?

### Solidification Kinetics as an Evolutionary Constraint.

The evolution of mineral morphologies is often discussed in terms of adaptation (98, 99) or environment (100, 101). However, the physical model presented here addresses the aspect of shell fabrication and can contribute to our understanding of the morphological evolution of molluscan shell ultrastructures within the framework of Seilacher’s constructional morphology (102) or morphodynamics (103). Here, the realization of biological forms is a product of the intersection of several constraining forces: Phylogenetic history, environment, function, and fabrication. As discussed, phylogeny appears to be a weak constraint as emphasized by the lack of a conserved genetic toolkit involved in mineral morphogenesis and the independent origin and repeated loss of ultrastructures, such as nacre (15, 96, 98). The external environment appears to influence mineral morphology, as illustrated by the connection between water temperature and pressure on the thickness of nacre tablets seen in both modern and fossil molluscs (86, 104, 105). Astonishingly, extreme changes in water temperature were even shown to lead to the formation of a completely different shell ultrastructure that is typically not produced by the organism (106). Furthermore, ocean chemistry has been suggested to have a role in polymorph selection during early clade evolution as well as a role in changing the dominant calcium carbonate polymorph (100, 107, 108). Function presents a clear and important role: A shell that is unable to resist fracture or boring is unlikely to persist through geologic time. The mechanics of different ultrastructures have long been the topic of extensive research and these biomaterials have been noted for their exceptional properties, such as the combination of high fracture resistance and stiffness (109). Thus, the convergent evolution of nacre in several molluscan clades may reflect a common solution to predator/prey escalation. However, it begs the question as to whether this convergence is due to nacre being the optimal morphology to resist such predation.

In Raup’s classic works, elegant parameterization of the macroscale geometry of molluscan shells defined a morphospace that permitted a theoretical exploration of the wide diversity of shell morphologies (110, 111). This work allowed a mathematical analysis of molluscan shell shapes and the theoretical exploration of limits of their evolution and thus, provided insights on why only certain morphologies exist in nature, in molluscs, and other species. For example, the similar spiral morphology seen in receptaculitids and bryozoans, despite very different methods of growth, reflects an architectural constraint on the fabrication of a growing spiral with physically constrained units (112).

In the present study, we extended a recently developed physical framework, which is based on an analogy to the process of directional solidification, to describe molluscan shell ultrastructural morphogenesis in species from 3 major classes. We demonstrate that the fabrication of these biocomposites is guided by the organisms by regulating the physical and chemical boundary conditions of the solidifying medium and, thus, controlling the growth kinetics of the mineral phase. In fact, we show that by the use of notions from classic materials science, Raup’s concept of morphospace can be expanded to the level of the ultrastructure. In turn, this can provide us with a unique opportunity to explore this morphospace using well-developed analytical, theoretical, and numerical tools and to test the effects of a discrete number of parameters, such as the abovementioned influence of temperature and pressure, on mineral morphology.

Ultimately, we suggest that the repeated “discovery” of mineral morphologies partially reflects a series of architectural constraints provided by the biomineral growth kinetics. Therefore, the convergent evolution of nacre is not a matter of whether nacre is an optimal structure to resist predation but whether nacre is a functionally optimal structure in a morphospace necessarily bounded by the thermodynamics and kinetics of crystal growth.

### Molluscan Shells and Beyond.

Although the 3 considered shells belong to different taxonomic classes, the investigated ultrastructures do not represent all of the possible structural motifs found in molluscs. These organisms were chosen due to our ability to fully analyze the morphological and crystallographic properties of the relatively coarse mineral building units and the gradual transition between the various ultrastructures that comprise their aragonitic shells. Other assemblies, such as the abundant crossed lamellar structure, and the various calcitic architectures are not discussed in this work. Therefore, while our analysis clearly supports the postulated biomineral morphogenesis mechanisms, additional experimental, analytical, and theoretical work is necessary to generalize the concepts presented in this study. Particularly, the development of an experimental framework to attain a correlation between morphological transitions within the shell with changes in the chemistry of the extrapallial fluid and the secretory regime of the epithelium inside a living organism will serve to evaluate the proposed model. Whereas this type of a physiological study was never previously reported, methods to extract the extrapallial fluid from living molluscs are well established (113).

Furthermore, molluscan shell biomineralization and morphogenesis is an extracellular process that proceeds under genetic control and is remotely orchestrated by the cellular tissue. The physical model developed here describes how this control is executed: By generating a driving force for mineral nucleation and growth in the form of biochemical and physical boundary conditions that guide the self-assembly of a specific morphology. However, mineral formation must adhere to the basic principles of crystal growth kinetics and thermodynamics regardless of whether it occurs extracellularly or within cells. Therefore, we believe that the introduced scientific approach is comprehensive. The main distinction between the growth of the various biomineralized structures in nature are the driving forces set by the organisms that ultimately regulate the nucleation, manipulate the shape, and assemble the mineral components. In this work, we demonstrate that identifying these forces is not only key to the study of biomineral formation, but is essential to our understanding of the most fundamental processes in evolution.

## Methods

### Sample Preparation.

Samples of the shells of *N. pompilius* and *H. asinina* were manually fractured parallel to the growth direction of the shell and coated with Pt/Pd for electron microscopy. For EBSD and AFM investigations, pieces of the shells of *N. pompilius* and *H. asinina* were embedded in poly(methyl methacrylate), cut parallel to the direction of growth, polished with a diamond solution, and finally polished with a colloidal silica solution.

### Electron Microscopy.

Imaging of the fractured and Pt/Pd-coated samples was performed using a Scios Dual Beam FIB/SEM (FEI/Thermo Fisher) in high-vacuum conditions.

### EBSD Analysis.

EBSD data were collected using an EDAX Hikari Super EBSD system on a Scios Dual Beam FIB/SEM (FEI/Thermo Fisher). To minimize damage to the specimen surface by the electron beam, we used a low current of 1.6 nA and a voltage of 15 kV. EBSD patterns were processed using neighbor pattern averaging indexing.

### AFM.

AFM measurements were performed in tapping mode using a JPK/Bruker NanoWizard4 AFM in combination with a Zeiss fluorescence microscope Axio Obersver Z1. A NANOSENSORS PointProbe Plus silicon probe (PPP-NCH) for tapping/noncontact mode with a typical tip radius of less than 7 nm and a spring constant of 42 N/m was used. The AFM measurements were performed using scan rates between 0.2 and 0.5 Hz.

### Phase-Field Modeling.

In the eutectic model applied here (81), the local state is characterized by 3 fields: 1) A space and time-dependent phase field ϕ(**r**,*t*) that monitors the solidification of the liquid, and is ϕ = 0 in the liquid, and ϕ = 1 in the solid (Movie S1); 2) a concentration field *c*(**r**,*t*) representing the local concentration of the organic matter (Movie S2); and 3) an orientation field θ(**r**,*t*), which specifies the local crystallographic orientation (a scalar field in 2D) (Movie S3). For brevity, in *SI Appendix* we present only a short summary of the model, which includes the free energy functional and the equations of motion. Further information is available in detail in Lewis et al. (81).

## Supplementary Material

Supplementary File

Supplementary File

Supplementary File

Supplementary File
