# Supplementary material for: Crystal growth kinetics as an architectural constraint on the evolution of molluscan shells
Source: Proc Natl Acad Sci U S A. 2019 Sep 24;116(41):20388–97. doi: 10.1073/pnas.1907229116 (PMC6789867; doi:10.1073/pnas.1907229116)
Supplement: Supplementary File [file pnas.1907229116.sapp.pdf]

PNAS
www.pnas.org

Supporting Information for

Crystal growth kinetics as an architectural constraint on the evolution of molluscan shells

Vanessa Schoeppler, Robert Lemanis, Elke Reich, Tamás Pusztai, László Gránásy and Igor Zlotnikov

Igor Zlotnikov
Email: igor.zlotnikov@tu-dresden.de

This PDF file includes:

Captions for movies S1 to S3
Summary of the Phase-Field Modeling Approach

Other supporting materials for this manuscript include the following:

Movies S1 to S3

www.pnas.org/cgi/doi/10.1073/pnas.1907229116

Captions for Movies S1 to S3:

Movie S1. The phase field map (white – solid, dark grey – liquid) during the directional solidification of a shell model as predicted by the orientation field based phase-field simulation under the same conditions as the simulation shown in Fig. 7, however, using a larger grid of 2000 × 1000. The white domain on the right represents the mantle. Note the fast solidification rate during the initial stages of shell formation.

Movie S2. The concentration field map of the mineral component (bright yellow denotes high concentration of the mineral component, whereas blue indicates the organic reach domains) during the directional solidification of a shell model as predicted by the orientation field based phase-field simulation under the same conditions as the simulation shown in Fig. 7, however, using a larger grid of 2000 × 1000. The black domain on the right represents the mantle.

Movie S3. The orientation field map (different colors stand for different crystallographic orientations) during the directional solidification of a shell model as predicted by the orientation field based phase-field simulation under the same conditions as the simulation shown in Fig. 7, however, using a larger grid of 2000 × 1000. The white domain on the right represents the mantle.

Summary of the Phase-Field Modeling Approach:

The free energy functional reads as:

$$F = \int d^3r \left\{ \frac{\varepsilon_\phi^{\ 2} T}{2} |\nabla\phi|^2 + \frac{\varepsilon_c^{\ 2} T}{2} |\nabla c|^2 + f_{bulk} + f_{ori} \right\}.$$

Here, the coefficient of the square-gradient terms for ϕ and c are $\varepsilon_\phi^2 = \varepsilon_c^2/6 = (12/\sqrt{2})\ \gamma_i\ \delta_i / T_i$ (i = A or B, which stand for the mineral and the organic components, respectively), where γ_i, δ_i, and T_i are the solid-liquid interface energy, the characteristic interface thickness, and the melting point for pure component i, respectively. The bulk free energy density was assumed to have the form $f_{bulk} = w(c)Tg(\phi) + [1 - p(\phi)]\, f_L(c,T) + p(\phi)\, f_S(c,T)$, where $w(c) = (1 - c)\ w_A + c\ w_B$, $w_i = (12/\sqrt{2})\ \gamma_i / (\delta_i T_i)$ (again, i = A or B), while $g(\phi) = ¼\ \phi^2(1 - \phi)^2$ and $p(\phi) = \phi^3(10 - 15\phi + 6\phi^2)$ are the double well and interpolation functions that determine the form of the free energy surface. These relationships originate from the works of Warren *et al.* (1) and Gránásy *et al.* (2). The contribution of the orientation field to the free energy density was assumed to have the form $f_{ori} = p(\phi) H\{h(c) F_1(|\nabla\theta|) + [1 - h(c)]\ F_2(|\nabla\theta|) + (\varepsilon_\theta^2 H/2T)|\nabla\theta|^2\}$ that keeps a fixed orientation-based relationship between the two solid phases (3). Here $h(c) = ½\{1 + \cos[2\pi\ (c - c_\alpha)/(c_\beta - c_\alpha)]\}$, c_α and c_β are the concentrations in the two solid phases, whereas $F_1(|\nabla\theta|) = |\nabla\theta|$ and $F_2(|\nabla\theta|) = a + b|\cos(2m\pi d|\nabla\theta)|$, while a, b, m, are constants, and d is the characteristic thickness of the α–β phase boundary. The equations of motion for the three fields were derived from $\partial\phi/\partial t = M_\phi\{\delta F/\delta\phi\}$, $\partial c/\partial t = \nabla[M_c \nabla\{\delta F/\delta c\}]$, and $\partial\theta/\partial t = M_\theta\{\delta F/\delta\theta\}$, where M_ϕ, M_c, and M_θ are the mobilities that determine the timescale of the evolution of the individual fields. The following model parameters were used: The equations of motion were made dimensionless using the characteristic length scale $\xi = 2.1\times10^{-6}$ m and time scale $\tau = \xi^2 / D_L$, expressed with the liquid diffusion coefficient $D_L = 10^{-9}$ m^2/s (2). Dimensionless spatial and time steps of $\Delta x = 6.25\times10^{-3}$ and $\Delta t = 9.5\times10^{-6}$ were used. The interface thicknesses were chosen as $\delta_i = 1.66\times10^{-7}$ m and $d = 1.31\times10^{-8}$ m, while $\gamma_B = 41.2$ mJ/m^2 and $\gamma_A = \gamma_B T_A/T_B$ were assumed. The used mobilities were $M_\phi = 0.0144\ D_L/(\varepsilon_\phi^2 T)$, $M_c = v_m D/(RT)\ c\ (1 - c)$, and $M_\theta = 12\ D_L/(\xi H)$ respectively. Here $D = [1 - p(\phi)]\ D_L$ and $H = 0.61\gamma_B$ were assumed. Other input data were taken from Lewis *et al.* (3), including the thermodynamic properties, and model parameters a, b, m, c_α, c_β, and the molar volume. We emphasize that these data may deviate

considerably from the (unknown) properties of the biomineralizing systems and were chosen to demonstrate that with appropriate model parameters/materials properties realistic microstructures can be obtained via directional solidification. The equations of motion were solved numerically on uniform rectangular grids of sizes 2000×200 and 2000×1000, using a finite difference scheme combined with explicit time stepping. The simulations were performed on a CPU cluster consisting of three blocks of 288, 192, and 128 cores, in which the nodes are connected by 40 Gbit/s Infiniband communication.

1. Warren JA, Boettinger WJ (1995) Prediction of dendritic growth and microsegregation patterns in a binary alloy using the phase-field method. *Acta Metall Mater* 43(2):689–703.
2. Gránásy L, Börzsönyi T, Pusztai T (2002) Nucleation and Bulk Crystallization in Binary Phase Field Theory. *Phys Rev Lett* 88(20):206105.
3. Lewis D, Warren J, Boettinger W, Pusztai T, Gránásy L (2004) Phase-field models for eutectic solidification. *JOM* 56(4):34–39.
